# Multimodal PA/US imaging and radiomics for the prediction of HER2-zero, -low, and -positive breast cancers: A novel approach for targeted therapy selection

**DOI:** 10.1016/j.pacs.2025.100764

**Published:** 2025-08-21

**Authors:** Zhibin Huang, Guoqiu Li, Mengyun Wang, Sijie Mo, Huaiyu Wu, Hongtian Tian, Shuzhen Tang, Jinfeng Xu, Fajin Dong

**Affiliations:** Department of Ultrasound, The Second Clinical Medical College, Jinan University (Shenzhen People's Hospital), Shenzhen, 518020, China

**Keywords:** Photoacoustic, HER2, Breast Cancer, Radiomics, Prediction

## Abstract

**Purpose:**

This study evaluates the efficacy of photoacoustic/ultrasound (PA/US) imaging-based radiomics for distinguishing HER2-zero, HER2-low, and HER2-positive breast cancer (BC), aiming to enhance targeted therapy selection.

**Methods:**

We analyzed 346 pathologically confirmed BC patients who underwent multimodal PA/US imaging at Shenzhen People’s Hospital from January 2022 to January 2025. HER2 status was determined pathologically and classified into three levels. Radiologists assessed conventional US features and manually segmented tumors on PA-images for radiomics feature extraction. Using the Least Absolute Shrinkage and Selection Operator analysis, we developed radiomics models for differentiating between HER2-zero versus HER2-low/positive cancers (Task 1), and HER2-low versus positive cancers (Task 2), and HER2-zero versus low cancers (Task 3). Patients were randomly divided into training sets and testing sets. Multivariate logistic regression was used to integrate radiomics, clinical-pathological, and US features into nomograms.

**Results:**

In testing set, radiomics features demonstrated an AUC of 0.846 with sensitivity of 79.3 % and specificity of 72.7 % for Task 1, and an AUC of 0.801 with sensitivity of 64.0 % and specificity of 82.8 % for Task 2, and an AUC of 0.767 with sensitivity of 80.7 % and specificity of 72.7 % for Task 3. For Task 1, 2 and 3, nomograms including PA imaging radiomics features combined with clinical-pathological features achieved AUCs of 0.848, 0.881 and 0.780, respectively.

**Conclusion:**

PA radiomics features effectively differentiate between HER2-zero and HER2 low/positive, and between HER2-low and HER2-positive BC, offering potential utility in guiding targeted therapy decisions.

**Summary:**

This study demonstrates the potential of PA imaging-based radiomics for accurately classifying HER2 expression statuses in BC, enhancing the selection process for targeted therapies. By integrating multi-modal imaging and pathology data, the developed radiomics models show robust performance, promising a non-invasive diagnostic supplementary for clinical application where traditional methods are limited.

## Highlights

• PA radiomics enables non-invasive distinction of HER2-zero, -low, and -positive breast cancers.
• Integrating PA radiomics with clinicopathological characteristics improves HER2 subtype classification accuracy.
• PA radiomics may guide HER2-targeted therapy, especially when IHC is inconclusive or FISH unavailable.

## 1 Introduction

Breast cancer (BC) is the predominant type of cancer and leading cause of cancer-related deaths among women [1]. Human Epidermal Growth Factor Receptor 2 (HER2) plays a pivotal role in the biology of BC, essential for prognostic assessment and as a crucial therapeutic target [2]. Traditionally, HER2 expression levels in BC have been determined using immunohistochemistry (IHC) and fluorescence in situ hybridization (FISH), with a binary classification system: HER2-negative (IHC 0, IHC 1 +, or IHC 2 + without FISH amplification) or HER2-positive (IHC 2 + with FISH amplification, or IHC 3 +) [3]. This conventional classification, however, limits the proportion of patients benefiting from targeted HER2 therapies [4], [5], [6], with HER2-negative tumors traditionally considered non-responsive [7], [8].

Recent revisions in classification further subdivide traditional HER2-negative cases into HER2-low (IHC 1 + or IHC 2 + without ISH amplification) and HER2-zero (IHC 0) statuses [8], [9]. Notably, HER2-low tumors, constituting more than half of all BC cases, exhibit unique biological characteristics, treatment response patterns, and clinical outcomes compared to other HER2 expression statuses [2], [4], [8], [10]. Emerging therapies, such as antibody-drug conjugates (ADCs), show promise in offering therapeutic benefits to patients with lower HER2 expression levels, not just those with overexpression [2], [8], [9]. With the recognition of HER2-low BC as a distinct entity, there is now a critical need for a ternary classification of HER2 expression - overexpressed, low, and zero - to accurately triage new BC diagnoses for appropriate anti-HER2 therapies [11].

Preoperative determination of HER2 status typically relies on pathological analysis of biopsy or surgical specimens. However, visual pathology assessments are susceptible to observer variability and the inherent heterogeneity of BC [12], with limited biopsy samples potentially not representing the entire tumor's biological information [13], [14]. Additionally, genetic analyses from pre-treatment biopsies capture the tumor biology at a single time point, which may evolve during and post-treatment [15]. The reliance solely on core needle biopsies has been inadequate, as studies have shown a misdiagnosis rate of approximately 20 % for HER2-low cases [16]. Thus, there is an urgent need for a simple, practical, and effective method to assess HER2 expression status pre-treatment.

Radiomics, the extraction of high-throughput features reflecting tissue heterogeneity from medical images, offers a repeatable, non-invasive marker to aid management where pathology results are ambiguous or biopsy procedures are limited [17], [18]. While ultrasound (US) radiomics has shown potential in inferring molecular states of BC, including using the traditional binary classification and new three-level classification of HER2 status [19], [20], [21], its diagnostic performance has been hampered by intrinsic limitations, such as the lack of functional information and overlapping morphological features of breast masses [22], [23]. Therefore, developing innovative tools that provide additional information to enhance the accuracy of US in assessing different HER2 statuses would be beneficial.

Photoacoustic (PA) imaging, when combined with conventional ultrasonography, offers a synergistic diagnostic approach to address several existing challenges in assessing breast lesions. By integrating laser technology with US, PA/US imaging not only provides detailed morphological insights but also functional data, such as imaging of the tumor vascular system and quantification of oxy- and deoxyhemoglobin levels, thereby enhancing diagnostic accuracy [24], [25], [26], [27], [28]. Recent studies indicate that PA imaging-based features correlate with the benign or malignant nature of breast lesions, providing a new perspective for diagnosis [29], [30]. However, there is still a lack of research successfully utilizing a precise method to diagnose HER2-low BC and comprehensive PA imaging analysis to characterize HER2-low BC. Thus, there is an urgent need to establish a non-invasive, user-friendly multimodal PA/US approach to accurately predict the HER2-low expression in BC patients.

The aim of this study is to evaluate the performance of PA imaging radiomics models in triaging the HER2 expression status of BC patients into three categories: HER2-positive, HER2-low, and HER2-zero. Our research seeks to use PA-based radiomics to preoperatively predict HER2 status, thereby assisting in the selection of candidates for traditional and novel HER2-targeted therapies and identifying individuals who could avoid unnecessary anti-HER2 treatment.

## 2 Materials and methods

### 2.1 Study population

**Fig. 1:**
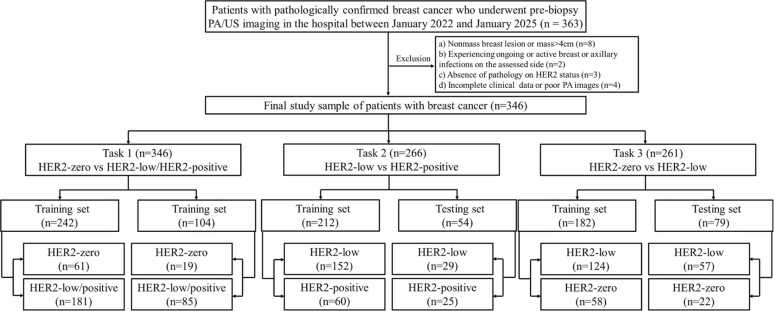
Flowchart of patient inclusion and exclusion criteria.

This study adhered strictly to the ethical standards set forth in the Declaration of Helsinki and received approval from the Medical Ethics Committee of our hospital (Approval Number: SYL-202161–02). Written informed consent was obtained from all participants. From January 2022 to January 2025, 306 patients with pathologically confirmed BC were included in this study, all of whom underwent multimodal PA/US breast examinations. The exclusion criteria were as follows: (1) pregnant or lactating women; (2) patients who had received neoadjuvant chemotherapy or interventional treatment for BC; (3) incomplete pathological information regarding HER2 status from IHC staining and/or FISH; (4) incomplete breast tissue in the region of interest (ROI); and (5) poor-quality PA images. Fig. 1 presents a flowchart of the patient inclusion process.

The baseline data for this study were collected from patient records, including demographic information (age, height, weight), BI-RADS classification, menopausal status, clinical T stage, tumor size, history of BC, histological grade, histological type, axillary ultrasound (AUS) status. Color Doppler Flow Imaging (CDFI) was routinely performed during ultrasound examinations to assess tumor vascularity and was graded semi-quantitatively using the Adler classification system (Grade 0-III) by experienced radiologists [31]. These categorical CDFI assessments were recorded as clinical imaging features and incorporated directly into the model, rather than being included in the radiomics pipeline. Immunohistochemistry (IHC) tests were used to determine the status of HER2, progesterone receptor (PR), estrogen receptor (ER), and Ki-67. Additionally, based on immunohistochemical staining and fluorescence in situ hybridization (FISH) results, the HER expression for each patient was classified into three categories: HER2-zero (IHC 0), HER2-low (IHC 1 + or IHC 2 + with a negative FISH result), and HER2-positive (IHC 2 + with a positive FISH result or IHC 3 +).

### 2.2 Dataset division

The division of the dataset aligns with the research tasks. In this study, we designed three classification tasks: Task 1 aims to distinguish between HER2-zero BC and HER2-low as well as HER2-positive BC; Task 2 aims to distinguish between HER2-low BC and HER2-positive BC; Task 3 aims to distinguish between HER2-zero BC and HER2-low BC. For each task, the study subjects were randomly assigned to the training set and testing set in a 7:3 ratio (each task was conducted separately). In Task 1, a total of 346 patients were collected, of which 242 were assigned to the training set [61 with HER2-zero BC and 181 with HER2-low or HER2-positive BC], and 104 were assigned to the testing set [19 with HER2-zero BC and 85 with HER2-low or HER2-positive BC]. In Task 2, out of 266 HER2-low or HER2-positive BC patients, 212 were assigned to the training set (152 HER2-low BC patients and 60 HER2-positive BC patients), and 54 were assigned to the testing set (29 HER2-low BC patients and 25 HER2-positive BC patients). In Task 3, out of 261 HER2-zero or HER2-low BC patients, 182 were assigned to the training set (124 HER2-low BC patients and 58 HER2-zero BC patients), and 79 were assigned to the testing set (57 HER2-low BC patients and 22 HER2-zero BC patients).

### 2.3 PA/US examination

**Fig. 2:**
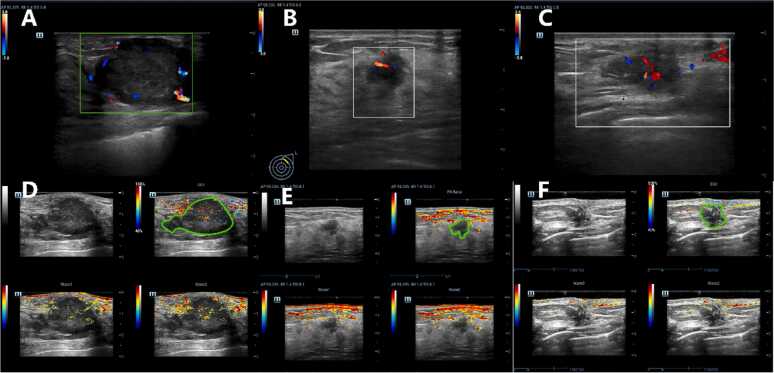
Multimodal Photoacoustic/ultrasound (PA/US) images of breast cancers with different HER2 expression levels. Note: Each panel displays a fused PA/US image, grayscale ultrasound (GSUS), and color Doppler flow imaging (CDFI). A manually segmented region of interest (ROI) is outlined to delineate tumor boundaries. Figure (A+D) displays a typical case of a HER2-positive breast cancer, irregular shape, parallel orientation, indistinct margins with microlobulations and spiculations, hypoechoic internal echo, posterior acoustic enhancement, architectural distortion, and obvious peripheral blood flow signals on CDFI. PA/US fusion images demonstrate intense red pseudocolor signals in both the intratumoral and peritumoral regions; Figure (B+E) presents a representative HER2-low case, irregular shape, non-parallel orientation, indistinct margins with microlobulations and angular features, hypoechoic internal echo, no posterior acoustic change, moderate internal blood flow signals on CDFI. PA/US fusion images reveal moderate red pseudocolor signals, predominantly at the tumor periphery; Figure (C+F) illustrates a HER2-zero lesion, irregular shape, non-parallel orientation, indistinct angular margins, hypoechoic internal echo, no posterior acoustic change, and obvious internal vascularity on CDFI. PA/US images demonstrate sparse blue pseudocolor signals, with low-intensity signals both within and around the tumor. The sO₂ maps presented in Fig. 2 were included for illustrative purposes only and were not quantitatively extracted or incorporated into the radiomics feature set. All features used for model development were derived from fused PA/US structural images, excluding functional parameters such as sO₂ and total hemoglobin (tHb). In the multimodal mode, the real-time imaging screen was divided into four segments. The top-left quadrant presented a standard US image, permitting the selection of grayscale ultrasound (GSUS), color Doppler US, or Power Doppler US. The bottom two quadrants displayed photoacoustic images superimposed on GSUS images at wavelengths of 750 nm (Wave 1) and 830 nm (Wave 2), respectively. The top-right quadrant showcased oxygen saturation (sO₂) mapping in pseudocolor, representing oxygenation derived from the combined signals of the two photoacoustic images at 750 nm and 830 nm.

The PA/US examination was conducted by one US physicians with 10 years of experience in US diagnosis, using a multimodal PA/US imaging system. This system was built on a commercial machine developed by Mindray Bio-Medical Electronics, equipped with a tunable optical parametric oscillator laser (InnoLas Laser) and a handheld linear PA/US probe L9–3. PA imaging was performed at two wavelengths, 750 nm and 830 nm, to measure oxygen saturation (sO₂). The resulting PA sO₂ (PA- sO₂) images were pseudocolorized and overlaid on the US images of the PA/US system (Fig. 2). Red and blue pixels represented high and low sO₂ values, respectively (imaging settings detailed in supplementary materials Appendix E1).

**Fig. 3:**
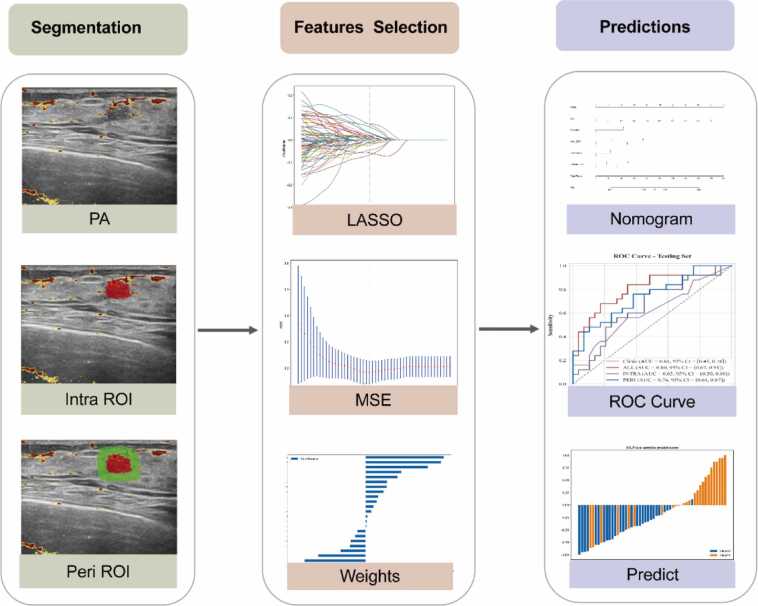
Workflow of radiomics and model construction.Note: PA: Photoacoustic; US: ultrasound; Intra: intratumoral; Peri: peritumoral; LASSO, least absolute shrinkage and selection operator algorithm; ROI: Region of Interest.

First, the patient raised both arms close to their head in a supine position to fully expose the imaging area. The radiologist scanned the entire breast using grayscale US to determine the location and the largest cross-section of the lesion. PA imaging was then performed on the largest cross-section of the lesion to obtain PA images and videos. During the examination, a coupling pad was placed between the probe and the breast for clear images. After the examination, the videos were saved to the hard drive of the imaging device for subsequent image analysis. The imaging depth range of the photoacoustic modality used in this study was approximately 0–4 cm. During the initial quality control process, any cases with tumors located beyond this effective depth were excluded. Tumor depth was defined as the vertical distance from the skin surface to the tumor deepest on grayscale ultrasound images. A summary of tumor depth distribution for the included cases is presented in Supplementary Figure S3.

### 2.4 Lesion segmentation

The collected PA/US images were in JPG format and were converted to nii.gz format using Python software. Two radiologists with 3 and 5 years of experience in breast US diagnosis, respectively, manually delineated the tumor edges on the PA/US images using ITK-SNAP software (www.itksnap.org), completing the segmentation of the ROI within the tumor. To ensure consistency in peritumoral ROI definition across patients, a fixed 5-mm ring was automatically generated around the manually segmented tumor boundary using a custom Python-based workflow. The segmentation was performed on the largest axial slice of the lesion, selected based on maximum tumor diameter. While tumor size influenced the extent of the intratumoral region, the peritumoral expansion was applied uniformly from the consensus-defined contours, enabling standardized extraction of peritumoral features independent of tumor size. Each reader performed the segmentation independently. The delineations were then reviewed jointly, and a consensus ROI was established. In cases of disagreement, the radiologists consulted with a senior expert with more than 10 years of experience in breast ultrasound imaging, and their judgment was used as the final reference.

### 2.5 Radiomics feature extraction and feature selection

Radiomics features were extracted using the PyRadiomics Python package. A total of 3122 quantitative features (intratumoral and peritumoral) were extracted from each ROI, namely the intratumoral region and the peritumoral region, on the PA/US fused images, rather than from isolated grayscale ultrasound or PA images. Extracted features were categorized into seven classes, including: (1) First-order statistics, which characterize the distribution of voxel intensities within the ROI; (2) Shape-based features (2D), which quantify tumor morphology and geometric complexity; (3) Gray Level Co-occurrence Matrix (GLCM) features, which assess spatial relationships between voxel intensities; (4) Gray Level Run Length Matrix (GLRLM) features, which measure the length of consecutive voxels with identical intensity; (5) Gray Level Size Zone Matrix (GLSZM) features, which evaluate the size of homogeneous intensity zones; (6) Gray Level Dependence Matrix (GLDM) features, which quantify local gray-level dependencies; and (7) Neighboring Gray Tone Difference Matrix (NGTDM) features, which capture intensity variations between a voxel and its surrounding neighborhood. These feature classes provide complementary information regarding tumor shape, signal heterogeneity, and spatial organization, which may be associated with underlying histopathologic characteristics such as stromal heterogeneity, cellular architecture, and angiogenesis. Shape-based features have also been associated with tumor invasiveness and molecular subtype differentiation, including HER2 expression status. To ensure the extracted features had good consistency and reproducibility, this study used the intraclass correlation coefficient (ICC) for analysis. Thirty patients were randomly selected, and their features were independently extracted by two physicians. The same steps were repeated by one of the physicians two weeks later. An ICC > 0.8 indicated good consistency.

The radiomics feature selection which underwent a rigorous screening process. Feature normalization for each patient was achieved through Z-score and mean normalization techniques, effectively standardizing the data for subsequent analyses. we performed the Mann-Whitney *U* test on all radiomics features to identify statistically significant features, retaining only those with a p-value less than 0.05. Next, we used the Spearman rank correlation coefficient to evaluate the correlation between the selected features. For any pair of features with a Spearman correlation coefficient greater than 0.9, one feature was excluded to reduce redundancy. The retained feature was selected based on higher robustness, as quantified by its ICC derived from inter-observer reproducibility analysis. For the final variable selection and modeling stage, we used the Least Absolute Shrinkage and Selection Operator (LASSO) regression model on the training dataset. By adjusting the regularization parameter λ, we shrank all regression coefficients towards zero, setting many irrelevant feature coefficients exactly to zero. To determine the optimal value of λ, we performed 10-fold cross-validation, selecting the λ that minimized the cross-validation error. The features with non-zero coefficients were then used to fit the regression model and develop the radiomics score. The radiomics score for each patient was calculated as a linear combination of the retained features, weighted by their respective model coefficients. The selected features were then used to train the predictive model using logistic regression (LR). The final selected features for each classification task were included in Supplementary (Appendix E2), which provides the feature name, category, PyRadiomics definition, and a brief interpretation of its potential biological or physical relevance. These interpretations were based on current understanding of tumor morphology and signal heterogeneity in relation to HER2 expression.

### 2.6 Radiomics signature and nomogram development

To effectively predict HER2 expression status in BC, we developed two PA radiomics models based on a three-tier classification. The first model (Task1) focuses on differentiating HER2-zero from HER2-low/positive cancers, while the second (Task2) distinguishes between HER2-low and HER2-positive cancers, and the third (Task3) distinguishes between HER2-zero and HER2-low cancers. These models were constructed by employing a LASSO regression method to select and weight the most relevant radiomics features. In parallel, we refined our approach to incorporating clinical data by initially using univariate logistic regression to identify significant predictors from the collected clinical data (P < 0.05). Subsequently, multivariate logistic regression was applied to these significant features to isolate independent predictors for model inclusion (P < 0.05). Finally, based on these features, a clinical prediction model was constructed using multivariate logistic regression.

Building on these foundations, we developed a clinical-radiomics nomogram. This tool integrates the refined radiomics scores with the independently significant clinical features through a multivariate logistic regression framework. The integration of these datasets into a single nomogram allows for an intuitive visualization, enhancing its utility in clinical practice by providing a straightforward method for predicting patient-specific HER2 status. This approach not only streamlines the diagnostic process but also advances personalized medicine by enabling precise treatment stratification based on quantifiable risk assessments.

### 2.7 Statistical analysis

Statistical analysis was performed using R version 3.8.0, with P < 0.05 considered statistically significant. First, the Shapiro–Wilk test was used to assess whether continuous variables followed a normal distribution. For continuous variables, the independent samples *t*-test or the Mann-Whitney *U* test was used; for categorical variables, the chi-square test or Fisher's exact test was used. Inter-observer and intra-observer consistency analyses were performed using intraclass correlation coefficients (ICCs). Univariate and multivariate analyses were performed using logistic regression models to identify clinically significant variables. Model performance was assessed by plotting receiver operating characteristic (ROC) curves and calculating the area under the curve (AUC), sensitivity, specificity, and accuracy.

## 3 Results

### 3.1 Clinicopathologic and US features

In this study, three distinct radiomics models were developed for tasks 1, 2 and 3, aimed at classifying BC into HER2-zero, -low, and -positive categories using PA radiomics. The clinical and pathological characteristics of patients in the training and testing sets for all three classification tasks are summarized in Table 1, Table 2, Table 3, while the US features are detailed in Supplementary Tables S1, S2 and S3.

**Table 1:** Clinicopathologic characteristics of patients with BC in each set for task 1.

Variables	Training set			Testing set		
	HER2-zero (*n* = 61)	HER2-low/positive (*n* = 181)	P	HER2-zero (*n* = 19)	HER2- low/positive (*n* = 85)	P
Age			0.404			1.000
≤40 year	11 (18)	44 (24)		3 (16)	13 (15)	
> 40 year	50 (82)	137 (76)		16 (84)	72 (85)	
Menopause			0.337			0.818
Premenopausal	36 (59)	92 (51)		11 (58)	44 (52)	
Postmenopausal	25 (41)	89 (49)		8 (42)	41 (48)	
Location			0.368			0.886
Left	31 (51)	78 (43)		9 (47)	36 (42)	
Right	30 (49)	103 (57)		10 (53)	49 (58)	
BIRADS			0.075			0.198
4 A	15 (25)	22 (12)		5 (26)	8 (9)	
4B	14 (23)	37 (20)		3 (16)	17 (20)	
4 C	26 (43)	91 (50)		7 (37)	46 (54)	
5	6 (10)	31 (17)		4 (21)	14 (16)	
Maximum diameter			0.706			0.891
≤20 mm	31 (51)	99 (55)		13 (68)	54 (64)	
> 20 mm	30 (49)	82 (45)		6 (32)	31 (36)	
History of breast cancer			1.000			0.021
Negative	53 (87)	159 (88)		19 (100)	66 (78)	
Positive	8 (13)	22 (12)		0 (0)	19 (22)	
HR			0.960			0.135
Negative	14 (23)	39 (22)		2 (11)	26 (31)	
Positive	47 (77)	142 (78)		17 (89)	59 (69)	
Ki67, n (%)			0.018			0.297
≤14	29 (48)	54 (30)		10 (53)	31 (36)	
> 14	32 (52)	127 (70)		9 (47)	54 (64)	
Histological grade			< 0.001			0.006
Ⅰ	20 (33)	22 (12)		9 (47)	12 (14)	
Ⅱ	21 (34)	99 (55)		5 (26)	46 (54)	
Ⅲ	20 (33)	60 (33)		5 (26)	27 (32)	
Clinical T stage			0.014			0.310
T1	36 (59)	72 (40)		12 (63)	40 (47)	
T2 or above	25 (41)	109 (60)		7 (37)	45 (53)	

Note. Except where indicated, data are numbers of women with percentages in parentheses. BI-RADS: Breast Imaging-Reporting and Data System; HER2: human epidermal growth factor receptor-2; Hormone receptor status (HR): estrogen receptor or progesterone receptor.

**Table 2:** Clinicopathologic characteristics of patients with BC in each set for task 2.

Variables	Training set			Testing set		
	HER2-low (*n* = 152)	HER2-positive (*n* = 60)	P	HER2-low (*n* = 29)	HER2-positive (*n* = 25)	P
Age	48 (40.75, 53)	52.5 (47.5, 58)	0.002	46.17 ± 6.99	51.44 ± 11.94	0.060
Menopause			0.185			0.029
Premenopausal	83 (55)	26 (43)		19 (66)	8 (32)	
Postmenopausal	69 (45)	34 (57)		10 (34)	17 (68)	
Location			0.751			0.190
Left	63 (41)	27 (45)		10 (34)	14 (56)	
Right	89 (59)	33 (55)		19 (66)	11 (44)	
BIRADS			0.032			0.037
4 A	14 (9)	9 (15)		2 (7)	5 (20)	
4B	38 (25)	8 (13)		7 (24)	1 (4)	
4 C	85 (56)	30 (50)		14 (48)	8 (32)	
5	15 (10)	13 (22)		6 (21)	11 (44)	
Maximum diameter			0.144			0.861
≤20 mm	92 (61)	29 (48)		18 (62)	14 (56)	
> 20 mm	60 (39)	31 (52)		11 (38)	11 (44)	
History of breast cancer			0.641			0.692
Negative	126 (83)	52 (87)		26 (90)	21 (84)	
Positive	26 (17)	8 (13)		3 (10)	4 (16)	
HR			< 0.001			0.026
Negative	19 (12)	33 (55)		3 (10)	10 (40)	
Positive	133 (88)	27 (45)		26 (90)	15 (60)	
Ki67			0.022			0.254
≤14	57 (38)	12 (20)		11 (38)	5 (20)	
> 14	95 (62)	48 (80)		18 (62)	20 (80)	
Histological grade			0.176			0.028
Ⅰ	17 (11)	5 (8)		8 (28)	4 (16)	
Ⅱ	94 (62)	31 (52)		14 (48)	6 (24)	
Ⅲ	41 (27)	24 (40)		7 (24)	15 (60)	
Clinical T stage			0.027			0.005
T1	70 (46)	17 (28)		19 (66)	6 (24)	
T2 or above	82 (54)	43 (72)		10 (34)	19 (76)	

Note. Except where indicated, data are numbers of women with percentages in parentheses. BI-RADS: Breast Imaging-Reporting and Data System; HER2: human epidermal growth factor receptor-2; Hormone receptor status (HR): estrogen receptor or progesterone receptor.

**Table 3:** Clinicopathologic characteristics of patients with BC in each set for task 3.

Variables	Training set			Testing set		
	HER2-zero (*n* = 58)	HER2-low (*n* = 124)	P	HER2-zero (*n* = 22)	HER2-low (*n* = 57)	p
Age			0.157			1.000
	10 (17)	35 (28)		4 (18)	10 (18)	
	48 (83)	89 (72)		18 (82)	47 (82)	
Menopause			0.380			0.457
Premenopausal	37 (64)	69 (56)		10 (45)	33 (58)	
Postmenopausal	21 (36)	55 (44)		12 (55)	24 (42)	
Location			0.199			0.875
Left	30 (52)	50 (40)		10 (45)	23 (40)	
Right	28 (48)	74 (60)		12 (55)	34 (60)	
BIRADS			0.004			0.793
4 A	16 (28)	10 (8)		4 (18)	6 (11)	
4B	13 (22)	35 (28)		4 (18)	10 (18)	
4 C	21 (36)	64 (52)		12 (55)	35 (61)	
5	8 (14)	15 (12)		2 (9)	6 (11)	
Maximum diameter			0.462			1.000
≤20 mm	31 (53)	75 (60)		13 (59)	35 (61)	
> 20 mm	27 (47)	49 (40)		9 (41)	22 (39)	
History of breast cancer			0.498			0.493
Negative	52 (90)	105 (85)		20 (91)	47 (82)	
Positive	6 (10)	19 (15)		2 (9)	10 (18)	
HR			0.396			0.274
Negative	11 (19)	16 (13)		5 (23)	6 (11)	
Positive	47 (81)	108 (87)		17 (77)	51 (89)	
Ki67			0.071			1.000
≤14	29 (50)	43 (35)		10 (45)	25 (44)	
> 14	29 (50)	81 (65)		12 (55)	32 (56)	
Histological grade			< 0.001			0.090
Ⅰ	21 (36)	16 (13)		8 (36)	9 (16)	
Ⅱ	18 (31)	74 (60)		8 (36)	34 (60)	
Ⅲ	19 (33)	34 (27)		6 (27)	14 (25)	
Clinical T stage			0.148			0.791
T1	35 (60)	59 (48)		13 (59)	30 (53)	
T2 or above	23 (40)	65 (52)		9 (41)	27 (47)	

Note. Except where indicated, data are numbers of women with percentages in parentheses. BI-RADS: Breast Imaging-Reporting and Data System; HER2: human epidermal growth factor receptor-2; Hormone receptor status (HR): estrogen receptor or progesterone receptor.

For Task 1 (HER2-zero vs HER2-low/HER2-positive), baseline demographic, clinicopathologic, and ultrasound characteristics of breast masses in the training and testing cohorts are summarized in Table 1 and Supplementary Table S1. In the training cohort, HER2-zero cancers differed significantly from HER2-low/HER2-positive cancers in Ki-67 index, histological grade, clinical T stage, and several ultrasound features, including presence of calcifications, external and internal color Doppler flow imaging (CDFI), internal echo pattern, and posterior acoustic features (all p < .05). In the testing cohort, significant differences were observed in history of breast cancer, histological grade, and the ultrasound features of orientation and internal CDFI (all p < .05).

For Task 2 (HER2-low vs HER2-positive), Table 2 and Supplementary Table S2 provide detailed summaries of baseline characteristics. In the training cohort, patients with HER2-low cancers showed significant differences from those with HER2-positive cancers in age, BI-RADS category, hormone receptor (HR) status, Ki-67 index, clinical T stage, and ultrasound features including AUS report, external and internal CDFI, lesion margin, and posterior echo (all p < .05). In the testing cohort, significant differences were noted in menopausal status, BI-RADS category, HR status, histological grade, and clinical T stage (all p < .05), whereas no ultrasound features showed significant differences (all p > .05).

For Task 3 (HER2-zero vs HER2-low), baseline features are presented in Table 3 and Supplementary Table S3. In the training cohort, HER2-zero and HER2-low cancers differed significantly in BI-RADS category, histological grade, and ultrasound characteristics including external and internal CDFI, lesion margin, and posterior acoustic features (all p < .05). In the testing cohort, no significant differences were found in clinicopathologic characteristics (all p > .05), while ultrasound features including orientation, external and internal CDFI, internal echo, and posterior echo showed significant differences between groups (all p < .05).

### 3.2 Model performance evaluation

#### 3.2.1 Task one: Differentiation of HER2-zero cancers from HER2-low or HER2-positive cancers

**Fig. 4:**
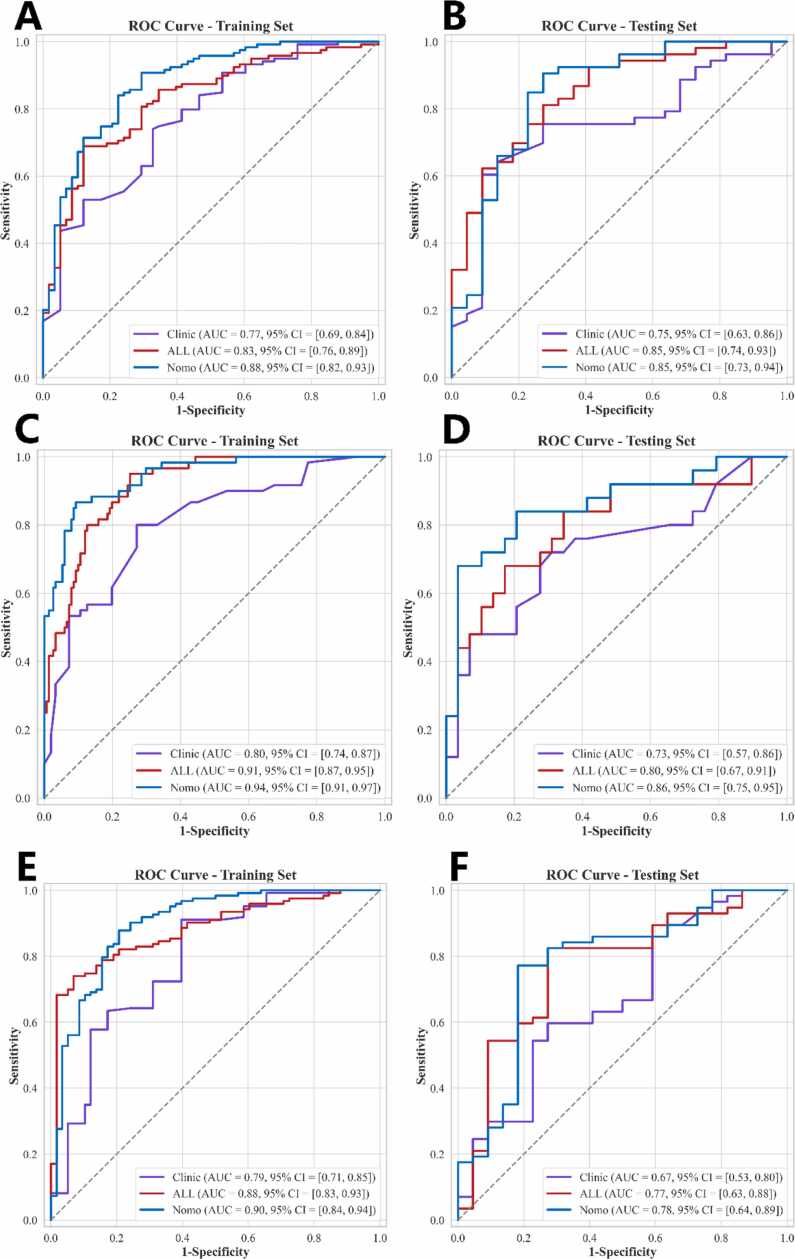
AUC curves for HER2-zero, -low, and -positive breast cancers. Note: A and B: AUC curves (Task 1) of the clinic model, ALL model and nomogram in the training set (A) and testing set (B). C and D: AUC curves (Task 2) of the clinic model, ALL model and nomogram in the training set (C) and testing set (D). E and F: AUC curves (Task 3) of the clinic model, ALL model and nomogram in the training set (C) and testing set (D). ALL: Radiomic signature within 5 mm of intratumoral + peritumoral. Nomo: nomogram, comprehensive model combining clinic and radiomic features. CI: confidence interval. AUC: area under the curve.

**Fig. 5:**
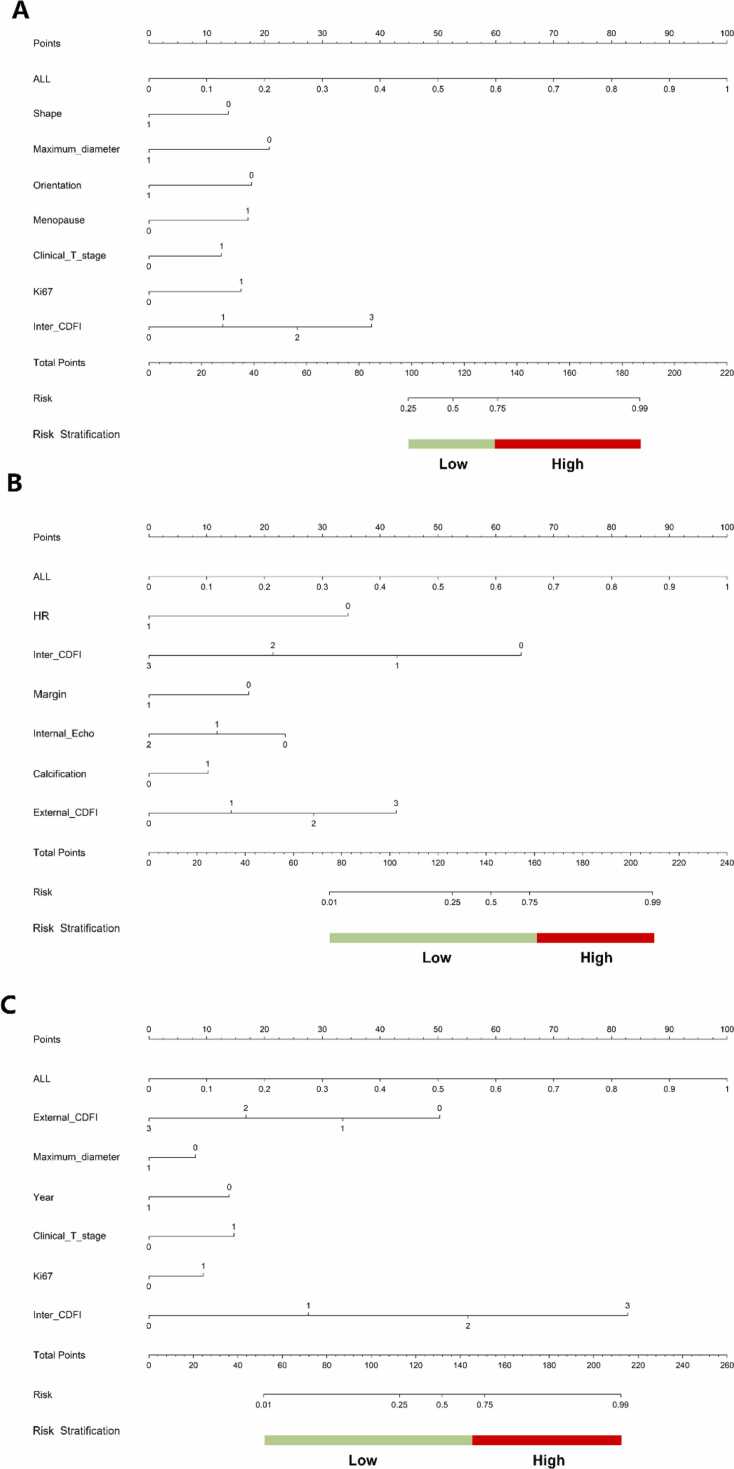
The construction of nomogram model. Note: A. (A) nomogram was developed incorporating the clinical features, intratumoral and peritumoral radiomic features for task1; B. (B) nomogram was developed incorporating the clinical features, intratumoral and peritumoral radiomic features for task2; C. (C) nomogram was developed incorporating the clinical features, intratumoral and peritumoral radiomic features for task3; ALL: Radiomic signature within intratumoral + 5 mm peritumoral. CDFI: color doppler flow imaging. Hormone receptor status: estrogen receptor or progesterone receptor; Ki67: 0 represents< 14, 1 represents≥ 14; internal Echo: 0 represents ultra-hypoechoic in the lesions, 1 represents hypoechoic, and 2 represents others; age: 0 represents ≤ 40 year, 1 represents > 40 year; Shape: 0 represents oval or round, 1 represents irregular; HR: 0 represents negative, 1 represents positive; maximum diameter: 0 represents ≤ 20 mm, 1 represents > 20 mm; orientation: 0 represents parallel, 1 represents not parallel; menopause: 0 represents premenopausal, 1 represents postmenopausal; menopause: 0 represents premenopausal, 1 represents postmenopausal; clinical T stage: 0 represents T1, 1 represents T2 or above; inter CDFI: 0 represents no, 1 represents little, 2 represents moderate, 3 represents obvious; external CDFI: 0 represents no, 1 represents little, 2 represents moderate, 3 represents obvious; margin: 0 represents circumscribed, 1 represents not circumscribed; calcification: 0 represents absent, 1 represents presence.

LASSO analysis identified 19 radiomic features for the importance of features is shown in Figure S2A. Of these, 8 features were extracted from within the tumor, while the remaining 11 were derived from the peritumoral area extending 5 mm from the tumor boundary. The formulas used to calculate these PA radiomics features are detailed in Table S4. When combining intratumoral and peritumoral features, the performance metrics in the testing set for identifying HER2-low or HER2-positive cancers included an AUC of 0.846 (95 % CI: 0.740–0.934), sensitivity of 79.3 %, specificity of 72.7 %, and an accuracy of 77.3 % (Table 4, Fig. 4A-B). Additionally, multivariate logistic regression analysis identified menopause, maximum diameter, orientation, shape, inter CDFI, Ki67 and Clinical T stage as independent predictors of HER2 status (Table 5). Nomograms incorporating PA radiomics and clinical-pathologic features were subsequently developed and are depicted in Fig. 5A. In the testing set, the nomogram that included clinical-pathologic features achieved an AUC of 0.848 (95 % CI: 0.726–0.945), with sensitivity and specificity of 88.7 % and 72.7 %, respectively (Table 4, Fig. 4A-B).

**Table 4:** Diagnostic performance of PA radiomics signature and PA radiomics nomogram in the three sets.

Model	Cohort	AUC	95 % CI	Sensitivity	Specificity	Accuracy	Precision
Task 1							
Clinical	Training	0.770	0.692–0.837	0.689	0.672	0.684	0.812
Intra+Peri-radiomics	Training	0.826	0.757–0.887	0.681	0.879	0.746	0.921
Nomogram	Training	0.878	0.818–0.927	0.832	0.776	0.814	0.884
Clinical	Testing	0.749	0.625–0.856	0.566	0.909	0.667	0.938
Intra+Peri-radiomics	Testing	0.846	0.740–0.934	0.793	0.727	0.773	0.875
Nomogram	Testing	0.848	0.726–0.945	0.887	0.727	0.840	0.887
Task 2							
Clinical	Training	0.795	0.729–0.861	0.617	0.828	0.768	0.587
Intra+Peri-radiomics	Training	0.914	0.877–0.951	0.933	0.748	0.801	0.596
Nomogram	Training	0.942	0.912–0.972	0.833	0.901	0.882	0.769
Clinical	Testing	0.761	0.635–0.888	0.56	0.828	0.704	0.737
Intra+Peri-radiomics	Testing	0.801	0.679–0.924	0.640	0.828	0.741	0.762
Nomogram	Testing	0.881	0.791–0.972	0.640	0.966	0.815	0.941
Task 3							
Clinical	Training	0.788	0.713–0.863	0.894	0.603	0.801	0.827
Intra+Peri-radiomics	Training	0.881	0.831–0.932	0.732	0.931	0.796	0.957
Nomogram	Training	0.893	0.838–0.948	0.886	0.776	0.851	0.893
Clinical	Testing	0.667	0.531–0.805	0.579	0.727	0.62	0.846
Intra+Peri-radiomics	Testing	0.767	0.645–0.890	0.807	0.727	0.785	0.885
Nomogram	Testing	0.780	0.657–0.903	0.737	0.818	0.759	0.913

Note: AUC, area under the curve; CI: confidence interval; intra, intratumoral features; peri, peritumoral features.

**Table 5:** Logistic regression analysis for identifying patients with HER2-zero BC, among patients with all BC (Task 1).

Characteristics	Univariate analysis			Multivariate analysis		
	OR	95 %CI	P value	OR	95 %CI	P value
Age	2.74	2.088–3.597	0.000	0.366	0.153–0.875	0.058
Menopause*	3.56	2.452–5.165	0.000	3.213	1.464–7.05	**0.015**
BIRADS	1.878	1.626–2.168	0.000	1.172	0.819–1.679	0.467
Maximum diameter*	2.733	1.925–3.881	0.000	0.263	0.118–0.587	**0.006**
Orientation*	2.000	1.326–3.016	0.006	0.274	0.114–0.658	**0.015**
History of breast cancer	2.750	1.395–5.425	0.014	1.870	0.676–5.176	0.312
Margin	3.255	2.474–4.284	0.000	2.844	1.129–7.164	0.063
Location	3.433	2.440–4.831	0.000	1.297	0.668–2.517	0.519
Shape*	2.847	2.221–3.651	0.000	0.155	0.036–0.664	**0.035**
Internal Echo	2.493	1.881–3.304	0.000	1.008	0.544–1.868	0.983
Posterior Echo	1.888	1.537–2.319	0.000	1.109	0.729–1.685	0.686
Inter CDFI*	2.190	1.835–2.614	0.000	4.902	2.23–10.773	**0.001**
External CDFI	1.949	1.669–2.275	0.000	0.498	0.235–1.057	0.127
AUS report	3.478	2.356–5.135	0.000	0.688	0.350–1.351	0.362
Calcification	5.200	3.480–7.768	0.000	1.552	0.717–3.357	0.349
HR	3.021	2.291–3.983	0.000	1.202	0.535–2.702	0.708
Ki67*	3.969	2.866–5.496	0.000	4.646	2.143–10.074	**0.001**
Clinical T stage*	4.360	3.028–6.278	0.000	3.830	1.675–8.758	**0.008**
Histological grade	1.666	1.487–1.866	0.000	0.962	0.552–1.679	0.909

Note. BI-RADS: Breast Imaging-Reporting and Data System; AUS report: axillary lymph nodes status reported by axillary ultrasound; CDFI: color doppler flow imaging; OR: Odd ratio; CI: Confidence interval; *: represents factors subsequently included in the nomogram model; Hormone receptor status (HR): estrogen receptor or progesterone receptor.

#### 3.2.2 Task two: Differentiation of HER2-low cancers from HER2-positive cancers

In Task 2, which aimed at distinguishing between HER2-positive and HER2-low cancers, LASSO analysis selected 23 radiomic features, comprising 12 from within the tumor and 11 from the peritumoral zone, as illustrated in Figures S2B. The computation of these PA radiomics features is detailed in Table S4, where the derived formulas for each feature are presented. When combining intratumoral and peritumoral features, the performance metrics in the testing set for identifying HER2-low from HER2-positive cancers included an AUC of 0.801 (95 % CI: 0.679–0.924), a sensitivity of 64.0 %, a specificity of 82.8 %, and an accuracy of 74.1 % (Table 4, Fig. 4C-D). Additionally, multivariate logistic regression analysis identified margin, internal echo, calcification, external CDFI, HR status and inter CDFI as independent predictors of HER2 status (Table 6). Nomograms incorporating PA radiomics and clinical-pathologic features were subsequently developed and are depicted in Fig. 5B. In the testing set, the nomogram that included clinical-pathologic features achieved an AUC of 0.881(95 % CI: 0.791–0.972), sensitivity of 64.0 %, and specificity of 96.6 % (Table 4, Fig. 4C-D).

**Table 6:** Logistic regression analysis for identifying patients with HER2-low BC, among patients with HER2-low or HER2-positive BC (Task 2).

Characteristics	Univariate analysis			Multivariate analysis		
	OR	95 %CI	P value	OR	95 %CI	P value
Age	0.456	0.346–0.600	0.000	1.505	0.561–4.035	0.496
Menopause	0.493	0.349–0.696	0.001	1.290	0.57–2.918	0.608
BIRADS	0.661	0.578–0.755	0.000	1.320	0.875–1.99	0.266
Maximum diameter	0.517	0.359–0.743	0.003	0.976	0.462–2.065	0.958
Orientation	0.222	0.125–0.395	0.000	0.450	0.185–1.099	0.141
History of breast cancer	0.308	0.158–0.598	0.004	0.620	0.222–1.733	0.444
Margin*	0.326	0.245–0.433	0.000	0.255	0.094–0.689	**0.024**
Location	0.371	0.265–0.518	0.000	0.792	0.398–1.573	0.576
Shape	0.389	0.300–0.504	0.000	3.000	0.773–11.635	0.182
Internal Echo*	0.480	0.356–0.647	0.000	0.444	0.234–0.844	**0.037**
Posterior Echo	0.617	0.497–0.766	0.000	1.153	0.744–1.786	0.594
Inter CDFI*	0.617	0.537–0.709	0.000	0.193	0.075–0.496	**0.004**
External CDFI*	0.641	0.561–0.733	0.000	3.485	1.383–8.776	**0.026**
AUS report	0.569	0.397–0.815	0.010	1.964	0.953–4.047	0.125
Calcification*	0.500	0.359–0.696	0.001	2.677	1.168–6.141	**0.048**
HR*	0.203	0.143–0.287	0.000	0.195	0.061–0.623	**0.021**
Ki67	0.505	0.378–0.676	0.000	1.056	0.470–2.370	0.911
Clinical T stage	0.524	0.385–0.715	0.001	2.751	1.131–6.693	0.061
Histological grade	0.694	0.621–0.774	0.000	0.748	0.395–1.418	0.455

Note. BI-RADS: Breast Imaging-Reporting and Data System; AUS report: axillary lymph nodes status reported by axillary ultrasound; CDFI: color doppler flow imaging OR: Odd ratio; CI: Confidence interval; *: represents factors subsequently included in the nomogram model; Hormone receptor status (HR): estrogen receptor or progesterone receptor.

#### 3.2.3 Task three: Differentiation of HER2-zero cancers from HER2- low cancers

In Task 3, which aimed at distinguishing between HER2-zero and HER2-low cancers, LASSO analysis selected 23 radiomic features, comprising 8 from within the tumor and 15 from the peritumoral zone, as illustrated in Figures S2C. The computation of these PA radiomics features is detailed in Table S4, where the derived formulas for each feature are presented. When combining intratumoral and peritumoral features, the performance metrics in the testing set for identifying HER2-low from HER2-positive cancers included an AUC of 0.767 (95 % CI: 0.645–0.890), a sensitivity of 80.7 %, a specificity of 72.7 %, and an accuracy of 78.5 % (Table 4, Fig. 4E-F). Additionally, multivariate logistic regression analysis identified age, maximum diameter, inter CDFI, external CDFI, Ki67 and Clinical T stage as independent predictors of HER2 status (Table 7). Nomograms incorporating PA radiomics and clinical-pathologic features were subsequently developed and are depicted in Fig. 5C. In the testing set, the nomogram that included clinical-pathologic features achieved an AUC of 0.780 (95 % CI:0.657–0.903), sensitivity of 73.7 %, and specificity of 81.8 % (Table 4, Fig. 4E-F).

**Table 7:** Logistic regression analysis for identifying patients with HER2-zero BC, among patients with HER2-low BC (Task 3).

Characteristics	Univariate analysis			Multivariate analysis		
	OR	95 %CI	P value	OR	95 %CI	P value
Age*	1.854	1.381–2.489	0.001	0.267	0.106–0.67	**0.018**
Menopause	2.619	1.718–3.995	0.000	2.224	0.942–5.249	0.126
BIRADS	1.611	1.379–1.881	0.000	1.026	0.658–1.598	0.925
Maximum diameter*	1.815	1.224–2.691	0.013	0.243	0.095–0.621	**0.013**
Orientation	1.773	1.143–2.748	0.032	0.413	0.166–1.027	0.111
History of breast cancer	3.167	1.465–6.841	0.014	2.621	0.872–7.877	0.150
Margin	2.391	1.791–3.193	0.000	2.734	0.892–8.373	0.140
Location	2.643	1.835–3.808	0.000	1.876	0.874–4.031	0.176
Shape	2.091	1.597–2.737	0.000	0.261	0.048–1.418	0.192
Internal Echo	1.881	1.374–2.575	0.001	0.971	0.472–1.994	0.946
Posterior Echo	1.467	1.179–1.826	0.004	0.938	0.563–1.564	0.836
Inter CDFI*	1.891	1.584–2.257	0.000	9.373	3.736–23.50	**0.000**
External CDFI*	1.642	1.409–1.914	0.000	0.232	0.092–0.590	**0.010**
AUS report	2.556	1.618–4.039	0.001	0.893	0.414–1.923	0.808
Calcification	3.158	2.048–4.87	0.000	0.687	0.277–1.704	0.497
HR	2.298	1.725–3.062	0.000	1.625	0.626–4.221	0.402
Ki67*	2.793	1.956–3.987	0.000	5.803	2.223–15.135	**0.003**
Clinical T stage*	2.826	1.896–4.212	0.000	3.922	1.539–9.994	**0.016**
Histological grade	1.447	1.280–1.637	0.000	0.929	0.479–1.804	0.855

Note. BI-RADS: Breast Imaging-Reporting and Data System; AUS report: axillary lymph nodes status reported by axillary ultrasound; CDFI: color doppler flow imaging OR: Odd ratio; CI: Confidence interval; *: represents factors subsequently included in the nomogram model; Hormone receptor status (HR): estrogen receptor or progesterone receptor.

## 4 Discussion

In this study, the diagnostic performance of PA radiomics was evaluated for stratifying HER2 expression into three clinically relevant categories. In the testing cohort, models based on combined intratumoral and peritumoral PA radiomics features yielded an AUC of 0.846 for distinguishing HER2-zero from HER2-low/HER2-positive cancers, 0.801 for distinguishing HER2-low from HER2-positive cancers, and 0.767 for differentiating HER2-zero from HER2-low cancers. Nomogram models incorporating PA radiomics features and pre-treatment clinicopathologic variables further improved classification performance. Specifically, the integrated nomogram achieved an AUC of 0.848 for differentiating HER2-zero from HER2-low/HER2-positive cancers, 0.881 for HER2-low versus HER2-positive cancers, and 0.780 for HER2-zero versus HER2-low cancers in the testing cohort. Importantly, these findings have clinical relevance given that different therapies may benefit patients with HER2-zero cancer (treatments other than HER2-targeted therapies), HER2-low cancer (novel anti-HER2 drugs), and HER2-positive cancer (traditional anti-HER2 medications). While the performance of PA radiomics alone may not support its use as a frontline test for determining HER2 expression status, these results suggest that PA radiomics could assist in assessing HER2 status in patients where HER2 expression is ambiguous with IHC staining or FISH analysis is unfeasible.

Previous studies have primarily focused on the traditional binary classification of HER2 expression in BC, examining imaging features for HER2-negative and positive cases, with varying diagnostic performance [32], [33], [34]. However, the emergence of new classification systems and novel ADC therapies has recently redirected attention towards previously overlooked HER2-low tumors [35]. HER2-low breast cancer, defined as IHC 1 + or 2 + with a negative FISH result, has traditionally been grouped under the HER2-negative category. However, recent clinical and molecular studies suggest that HER2-low tumors may represent a biologically distinct subgroup [36]. Notably, the Phase III DESTINY-Breast04 trial demonstrated that trastuzumab deruxtecan, a HER2-targeting antibody-drug conjugate (ADC), significantly prolonged progression-free and overall survival in patients with HER2-low metastatic breast cancer compared to standard chemotherapy [37]. This landmark finding has shifted the therapeutic landscape and renewed attention toward the importance of accurate HER2-low classification. Current research on the imaging morphology and radiomics of HER2-low tumors is expanding. For instance, Ramtohul et al. utilized multiparametric MRI radiomics to predict HER2-low status and performed subgroup analysis based on ISH scoring, achieving an AUC between 0.76 and 0.82, indicating strong performance [38]. Similarly, Peng et al. used multiparametric MRI radiomics features to differentiate HER2-zero from HER2-low or HER2-positive cancers, as well as HER2-low from HER2-positive cancers, with AUCs ranging from 0.78 to 0.89 and 0.75–0.77 for the two tasks respectively, demonstrating good diagnostic accuracy [39]. Despite these advancements, the high cost, long waiting times, and inapplicability to certain patients limit the widespread use of MRI. In contrast, US, a mainstay in clinical BC screening, offers the advantages of broader availability and cost-effectiveness. Recent studies [40] have shown that US radiomics can non-invasively distinguish between HER2-low/positive and HER2-zero cases with AUCs of 0.81–0.84, and further differentiate between HER2 1 + , HER2 2 + , and HER2-zero cases with AUCs from 0.79 to 0.87. However, inherent limitations of US, including the lack of functional information and overlapping morphological features of breast masses, have hindered its broad application [22], [23].

This study leverages multimodal PA/US imaging to produce time-staggered, co-registered real-time images. This system integrates Grayscale US with color-coded PA functionality of breast lesions, providing comprehensive visualization that enhances diagnostic accuracy and efficiency. Specifically, PA imaging offers high-resolution deep tissue imaging and insights into the molecular and functional aspects of cancer cells, working synergistically with US to provide multiparametric information about malignant tumors [28], [41]. Furthermore, unlike previous studies by Du et al. [40] that did not differentiate between HER2-positive and HER2-low cancers as done in Task 2 of this study, our approach helps inform potential HER2-targeted therapy decisions by applying quantitative PA imaging methods. Our findings indicate that PA imaging radiomics features were less effective in Task 1 than in Task 2, suggesting that differentiating between absent and expressed HER2 levels is more challenging than separating expressed HER2 levels. Although ISH and FISH remain the gold standards for HER2 expression assessment in clinical practice, these methods rely on independent biopsies of tumors with intrinsic heterogeneity, capturing only a fraction of the biological information [42]. The tumor's heterogeneity makes it challenging to ascertain HER2 expression through biopsy alone [43]. Therefore, the ISH and FISH are subject to technical variability and interpretation discrepancies, particularly near the IHC 0/1 + threshold. In this context, radiomics based on multimodal PA/US imaging offers a promising non-invasive strategy to complement diagnostic tool for pathologists, benefiting patients requiring different treatment regimes. By quantifying tumor morphology, texture, and vascular heterogeneity, PA/US radiomic features may assist in: Alerting clinicians to equivocal or borderline HER2 cases, guiding targeted biopsy of regions more likely to reflect HER2 expression, enabling longitudinal tracking of HER2 status during neoadjuvant or metastatic treatment. These capabilities may be particularly beneficial for triple-negative or hormone receptor–positive patients who might become candidates for ADCs upon HER2-low reclassification. Therefore, incorporating imaging-derived biomarkers into the clinical workflow may enhance HER2 subtype stratification and support more personalized treatment decisions.

Previous studies on radiomics primarily focused on intratumoral features in predicting low expression, overexpression, and absence of HER2 in BC, neglecting valuable clinical information present in the peritumoral area [38], [39], [40], [44]. However, features of the surrounding tissue are crucial in the decision-making process and should not be overlooked [45]. The presence of a fibroproliferative response or cancer cell infiltration into the interstitial tissues indicates that peritumoral changes can reflect the progression of aggressive malignant tumors [46]. Moreover, the tumor microenvironment plays a key role in tumor progression and metastasis, as the secretion of cytokines and growth factors can lead to hypoxia and angiogenesis [47]. In this study, by combining features from both peritumoral and intratumoral areas, our composite model maximized the assessment of HER2 expression status, consistently outperforming models that only included intratumoral features. This finding aligns with the work by Dong et al. [45], which demonstrated that diagnosing based solely on peripheral tissue is inadequate when lesions are present. Notably, models with an appropriate peritumoral area exhibited higher AUC values, as these areas more comprehensively characterized the tumor's heterogeneity and aggressiveness. However, as previous studies have shown [41], including a larger peritumoral area does not always enhance model performance, potentially because an excessively large peritumoral area may include normal tissue biology irrelevant to the diagnosis, confusing machine learning algorithms. Further research is needed to validate and elucidate these mechanisms. In conclusion, PA imaging-based radiomics holds promise as a potential predictor for HER2-low status. Model based on photoacoustic imaging demonstrated commendable diagnostic accuracy in differentiating between HER2 statuses. Our study represents a preliminary exploration into using PA radiomics to predict HER2-low status and perform subgroup analysis of HER2-zero, low, and positive tumors in a clinical setting. External testing suggests that PA imaging-based radiomics can effectively differentiate between HER2-low patients.

Notably, in Task 1, multivariable logistic regression analysis identified menopause, maximum diameter, orientation, shape, inter CDFI, Ki67 and Clinical T stage as independent predictors of HER2 status. In Task 2, multivariable logistic regression analysis identified margin, internal echo, calcification, external CDFI, HR status and inter CDFI emerged as significant predictors. In Task 3, multivariable logistic regression analysis identified age, maximum diameter, inter CDFI, external CDFI, Ki67 and Clinical T stage as independent predictors of HER2 status. While some studies have indicated that tumor posterior echo is independent predictors of HER2 expression status in BC, these associations were not apparent in our analysis [39], [40]. This discrepancy may stem from the fact that clinical parameters alone can only capture a limited snapshot of tumor characteristics. Additionally, previous research focusing on classifying low expression as negative has limited studies on this topic. Further research and discussion are needed to determine whether any imaging differences exist between different HER2 expression groupings.

While the results of this study are encouraging, several limitations warrant attention. Firstly, this was a single-center study lacking independent external validation, highlighting the need for future prospective, multicenter studies to further validate the model across diverse clinical settings. Secondly, the study included a small number of HER2-zero BC patients (n = 80), with only 19 such patients in the testing set. This calls for the inclusion of more HER2-zero patients in future research to enhance the robustness of the findings. Additionally, the delineation of the regions of interest (ROI) was performed manually, making the extraction of radiomics features both time-consuming and complex. Future efforts should aim to automate the segmentation process, particularly through the use of deep learning algorithms. What’s more, although our methodology expanded the initial ROI radially by 5 mm from the tumor margin based on findings from previous studies [48], future research should investigate the predictive value of different expansion distances around the tumor area in relation to HER2 expression levels. Lastly, only GSUS and CDFI were incorporated alongside photoacoustic radiomic features in the present study. Functional photoacoustic parameters such as tissue sO₂ and total hemoglobin concentration (HbT) were not included in the current analysis. Future studies integrating these functional metrics may further enhance subtype discrimination by providing complementary physiologic information beyond morphology- and texture-based features.

## 5 Conclusion

This study investigated the efficacy of PA imaging-based radiomics features for distinguishing HER2-zero BC from other HER2 expression statuses and further differentiating HER2-low from HER2-positive BC. By integrating intratumoral and peritumoral PA radiomics features with US imaging and breast pathology characteristics, the radiomics models demonstrated robust performance across both training and testing cohorts. The findings suggest that PA imaging-based radiomics models hold potential as a non-invasive tool for differentiating HER2 expression statuses in BC patients. This could assist in selecting candidates for novel or traditional HER2-targeted therapies, especially in cases where IHC results are inconclusive or FISH testing is unavailable.

## CRediT authorship contribution statement

**Sijie Mo:** Resources, Methodology. **Huaiyu Wu:** Resources, Investigation. **Hongtian Tian:** Resources, Data curation. **Shuzhen Tang:** Resources. **Zhibin Huang:** Writing – original draft, Resources, Methodology, Conceptualization. **Guoqiu Li:** Methodology, Formal analysis, Data curation. **Mengyun Wang:** Validation, Resources. **Jinfeng Xu:** Writing – review & editing, Supervision. **Fajin Dong:** Writing – review & editing, Supervision, Resources.

## Ethics approval and consent to participate

This study was approved by the Institutional Review Board of the Shenzhen People’s Hospital, specifically the Medical Ethics Committee of Shenzhen People 's Hospital. and all participants provided written informed consent. All methods were carried out in accordance with relevant guidelines and regulations.

## Financial support

No.

## Declaration of Competing Interest

The authors declare that they have no known competing financial interests or personal relationships that could have appeared to influence the work reported in this paper.

## Data Availability

Data will be made available on request.
