# Supplementary material for: Multimodal PA/US imaging and radiomics for the prediction of HER2-zero, -low, and -positive breast cancers: A novel approach for targeted therapy selection
Source: Photoacoustics. 2025 Aug 21;46:100764. doi: 10.1016/j.pacs.2025.100764 (PMC12446380; doi:10.1016/j.pacs.2025.100764)
Supplement: Supplementary file 1 — Supplementary material [file mmc1.docx]

**Appendix E1**

**Imaging Settings and Operating Procedures of the Multimodal PA/US**

**Imaging System**

The multimodal photoacoustic and ultrasound (PA/US) imaging system, depicted in Figures S1, provides resolutions finer than 1 mm in both horizontal and vertical dimensions and achieves a signal-to-noise ratio of 27.5 dB at depths ranging from 5 to 20 mm. It emits light on the tissue surface at a safe intensity of less than 20 mJ/cm², ensuring minimal fluctuations within a 5% range. To minimize photoacoustic noise, especially under bone areas, the system's gain settings were adjusted from 55 dB down to 45 dB. The Power Doppler US (PDUS) mode utilizes settings that include a pulse repetition frequency (PRF) of 600-1000 Hz, a wall filter from 50-100 Hz, a maximum gain of 85-90%, a velocity scale fixed at 3 cm/s, and a non-angled rectangular sampling box.


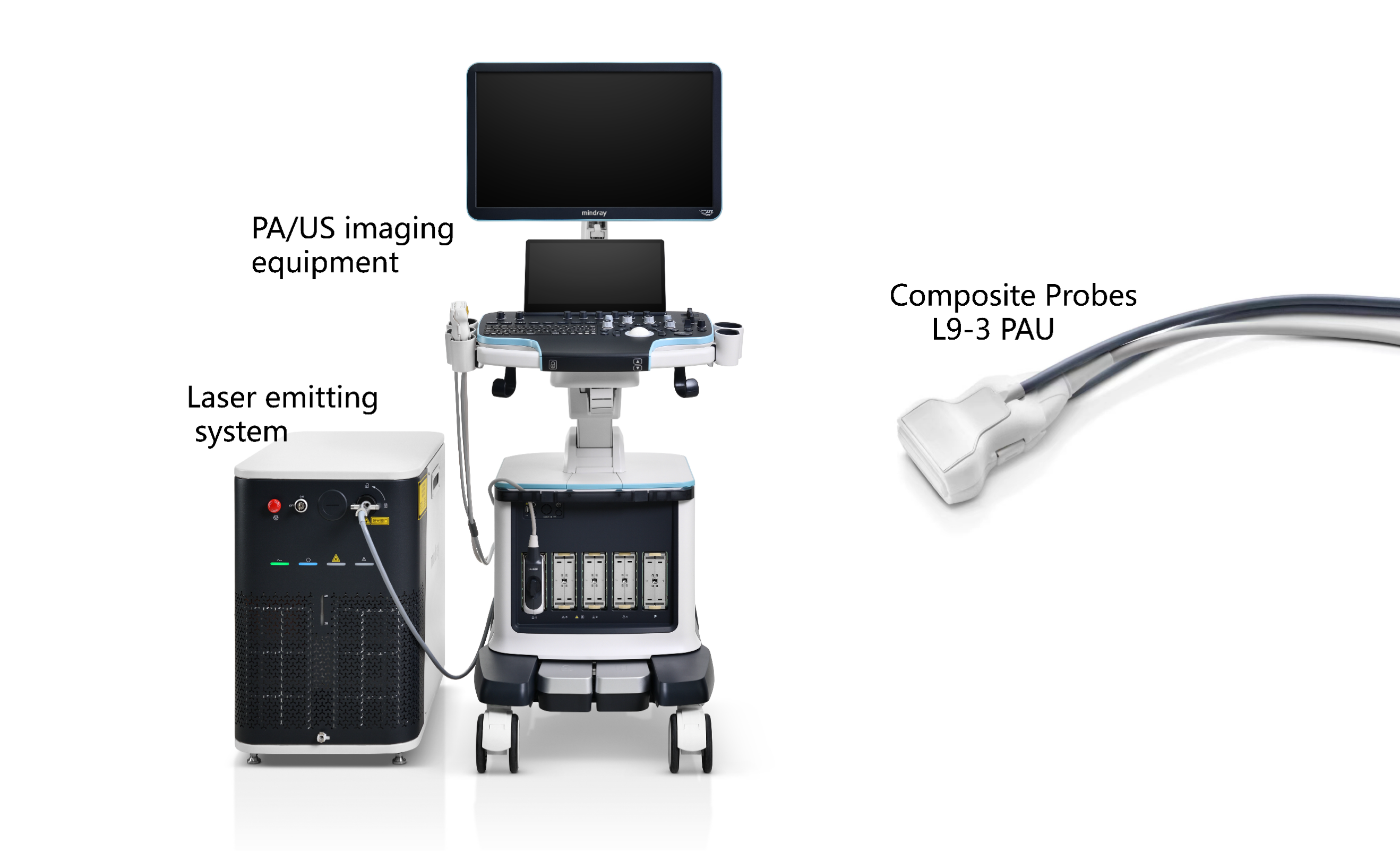


Figure S1: Photoacoustic/ultrasound imaging system and imaging probe (L9-3PAU, center frequency 5.5 MHz).

**
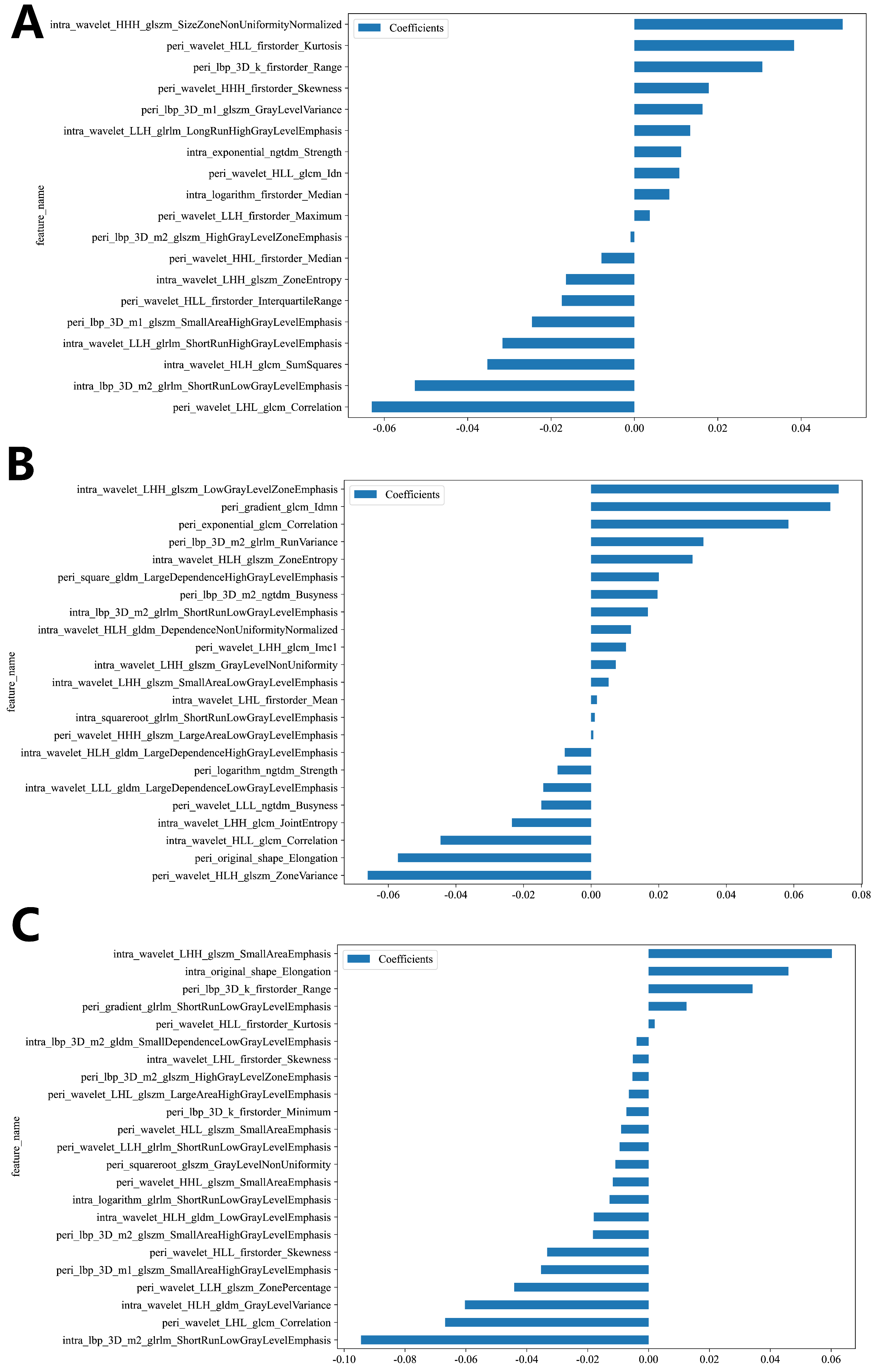
**

**Figure S2:** ALL feature weights. Note. A: intratumoral and peritumoral 5 mm region-ALL feature weights, LASSO regression was used to select the 19 best features intratumoral for constructing the model for Task 1; B: intratumoral and peritumoral 5 mm region-ALL feature weights, LASSO regression was used to select the 23 best features intratumoral for constructing the model for Task 2; C: intratumoral and peritumoral 5 mm region-ALL feature weights, LASSO regression was used to select the 23 best features intratumoral for constructing the model for Task 3. The negative values in the LASSO output refer to the coefficients of the predictors in our model. A positive value indicates a direct relationship between the feature and the response variable, suggesting that as the feature increases, the expected value of the response variable also increases. Conversely, a negative value indicates an inverse relationship, suggesting that as the feature increases, the expected value of the response variable decreases.


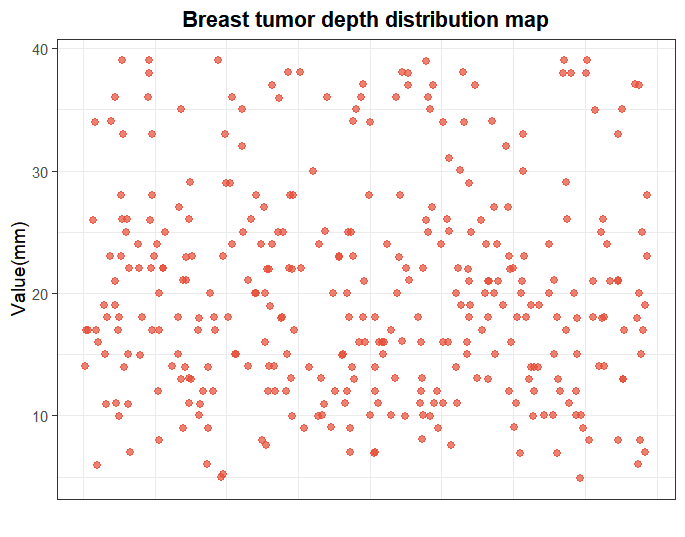


Figure S3. Distribution of tumor depths measured from the skin surface to the tumor deepest on grayscale ultrasound. Note. The scatter plot illustrates the distribution of tumor depth across all 346 included patients. Most lesions were located between 4.9 and 39 mm, confirming their suitability for PA imaging acquisition. All analyzed cases fell within the effective imaging depth range (0–4 cm) of the PA modality.

**Table S1.** Distribution of ultrasound features in Task 1.

| Variables | Training set | | | Testing set | | |
| --- | --- | --- | --- | --- | --- | --- |
|  | HER2-zero  (*n* = 61) | HER2-low/positive  (*n* = 181) | P | HER2-zero  (*n* = 19) | HER2-  low/positive  (*n* = 85) | P |
| AUS report |  |  | 0.461 |  |  | 0.394 |
| Unsuspicious | 38 (62) | 101 (56) |  | 14 (74) | 51 (60) |  |
| Suspicious | 23 (38) | 80 (44) |  | 5 (26) | 34 (40) |  |
| Calcification |  |  | 0.001 |  |  | 0.261 |
| Absent | 41 (67) | 77 (43) |  | 14 (74) | 48 (56) |  |
| Presence | 20 (33) | 104 (57) |  | 5 (26) | 37 (44) |  |
| Orientation |  |  | 0.083 |  |  | 0.015 |
| Parallel | 37 (61) | 133 (73) |  | 10 (53) | 69 (81) |  |
| Not parallel | 24 (39) | 48 (27) |  | 9 (47) | 16 (19) |  |
| External CDFI |  |  | < 0.001 |  |  | 0.101 |
| No | 9 (15) | 26 (14) |  | 4 (21) | 13 (15) |  |
| Little | 36 (59) | 66 (36) |  | 8 (42) | 30 (35) |  |
| Moderate | 10 (16) | 22 (12) |  | 4 (21) | 7 (8) |  |
| Obvious | 6 (10) | 67 (37) |  | 3 (16) | 35 (41) |  |
| Inter CDFI |  |  | < 0.001 |  |  | < 0.001 |
| No | 21 (34) | 27 (15) |  | 5 (26) | 17 (20) |  |
| Little | 26 (43) | 70 (39) |  | 10 (53) | 27 (32) |  |
| Moderate | 10 (16) | 18 (10) |  | 4 (21) | 7 (8) |  |
| Obvious | 4 (7) | 66 (36) |  | 0 (0) | 34 (40) |  |
| Shape |  |  | 0.368 |  |  | 0.636 |
| Oval or Round | 2 (3) | 13 (7) |  | 2 (11) | 6 (7) |  |
| irregular | 59 (97) | 168 (93) |  | 17 (89) | 79 (93) |  |
| Margin |  |  | 0.255 |  |  | 0.519 |
| Circumscribed | 14 (23) | 28 (15) |  | 5 (26) | 15 (18) |  |
| Not circumscribed | 47 (77) | 153 (85) |  | 14 (74) | 70 (82) |  |
| Internal Echo |  |  | 0.023 |  |  | 0.550 |
| Ultra-hypoechoic | 17 (28) | 71 (39) |  | 8 (42) | 45 (53) |  |
| Hypoechoic | 44 (72) | 100 (55) |  | 11 (58) | 39 (46) |  |
| Others | 0 (0) | 10 (6) |  | 0 (0) | 1 (1) |  |
| Posterior Echo |  |  | 0.021 |  |  | 0.090 |
| Shadowing | 17 (28) | 75 (41) |  | 4 (21) | 40 (47) |  |
| No posterior features | 30 (49) | 54 (30) |  | 10 (53) | 32 (38) |  |
| Enhancement | 14 (23) | 52 (29) |  | 5 (26) | 13 (15) |  |

Note. Except where indicated, data are numbers of women with percentages in parentheses. AUS report: BCs status reported by axillary ultrasound; CDFI: color doppler flow imaging.

**Table S2.** Distribution of ultrasound features in Task 2

| Variables | Training set | | | Testing set | | |
| --- | --- | --- | --- | --- | --- | --- |
|  | HER2-low  (*n* = 152) | HER2-positive  (*n* = 60) | P | HER2-low  (*n* = 29) | HER2-positive  (*n* = 25) | P |
| AUS report |  |  | 0.038 |  |  | 0.115 |
| Unsuspicious | 94 (62) | 27 (45) |  | 20 (69) | 11 (44) |  |
| Suspicious | 58 (38) | 33 (55) |  | 9 (31) | 14 (56) |  |
| Calcification |  |  | 0.121 |  |  | 1.000 |
| Absent | 78 (51) | 23 (38) |  | 13 (45) | 11 (44) |  |
| Presence | 74 (49) | 37 (62) |  | 16 (55) | 14 (56) |  |
| Orientation |  |  | 0.078 |  |  | 1.000 |
| Parallel | 107 (70) | 50 (83) |  | 24 (83) | 21 (84) |  |
| Not parallel | 45 (30) | 10 (17) |  | 5 (17) | 4 (16) |  |
| External CDFI |  |  | 0.008 |  |  | 0.399 |
| No | 26 (17) | 5 (8) |  | 4 (14) | 4 (16) |  |
| Little | 48 (32) | 34 (57) |  | 5 (17) | 9 (36) |  |
| Moderate | 18 (12) | 4 (7) |  | 5 (17) | 2 (8) |  |
| Obvious | 60 (39) | 17 (28) |  | 15 (52) | 10 (40) |  |
| Inter CDFI |  |  | 0.002 |  |  | 0.272 |
| No | 29 (19) | 7 (12) |  | 4 (14) | 4 (16) |  |
| Little | 45 (30) | 35 (58) |  | 6 (21) | 11 (44) |  |
| Moderate | 15 (10) | 4 (7) |  | 4 (14) | 2 (8) |  |
| Obvious | 63 (41) | 14 (23) |  | 15 (52) | 8 (32) |  |
| Shape |  |  | 0.744 |  |  | 1.000 |
| Oval or Round | 8 (5) | 4 (7) |  | 4 (14) | 3 (12) |  |
| Irregular | 144 (95) | 56 (93) |  | 25 (86) | 22 (88) |  |
| Margin |  |  | 0.010 |  |  | 0.485 |
| Circumscribed | 17 (11) | 16 (27) |  | 4 (14) | 6 (24) |  |
| Not Circumscribed | 135 (89) | 44 (73) |  | 25 (86) | 19 (76) |  |
| Internal Echo |  |  | 0.715 |  |  | 0.889 |
| Ultra-hypoechoic | 67 (44) | 23 (38) |  | 15 (52) | 11 (44) |  |
| Hypoechoic | 79 (52) | 34 (57) |  | 13 (45) | 13 (52) |  |
| Others | 6 (4) | 3 (5) |  | 1 (3) | 1 (4) |  |
| Posterior Echo |  |  | 0.039 |  |  | 0.997 |
| Shadowing | 72 (47) | 19 (32) |  | 13 (45) | 11 (44) |  |
| No posterior features | 42 (28) | 27 (45) |  | 9 (31) | 8 (32) |  |
| Enhancement | 38 (25) | 14 (23) |  | 7 (24) | 6 (24) |  |

Note. Except where indicated, data are numbers of women with percentages in parentheses. AUS report: BCs status reported by axillary ultrasound; CDFI: color doppler flow imaging.

**Table S3.** Distribution of ultrasound features in Task 3

| Variables | Training set | | | Testing set | | |
| --- | --- | --- | --- | --- | --- | --- |
|  | HER2-zero  (*n* = 58) | HER2-low  (*n* = 124) | P | HER2-zero  (*n* = 22) | HER2-low  (*n* = 57) | P |
| AUS report |  |  | 0.528 |  |  | 0.656 |
| Unsuspicious | 40 (69) | 78 (63) |  | 12 (55) | 36 (63) |  |
| Suspicious | 18 (31) | 46 (37) |  | 10 (45) | 21 (37) |  |
| Calcification |  |  | 0.068 |  |  | 0.076 |
| Absent | 39 (67) | 64 (52) |  | 16 (73) | 27 (47) |  |
| Presence | 19 (33) | 60 (48) |  | 6 (27) | 30 (53) |  |
| Orientation |  |  | 0.487 |  |  | 0.014 |
| Parallel | 36 (62) | 85 (69) |  | 11 (50) | 46 (81) |  |
| Not parallel | 22 (38) | 39 (31) |  | 11 (50) | 11 (19) |  |
| External CDFI |  |  | < 0.001 |  |  | 0.037 |
| No | 8 (14) | 19 (15) |  | 5 (23) | 11 (19) |  |
| Little | 34 (59) | 37 (30) |  | 10 (45) | 16 (28) |  |
| Moderate | 9 (16) | 16 (13) |  | 5 (23) | 7 (12) |  |
| Obvious | 7 (12) | 52 (42) |  | 2 (9) | 23 (40) |  |
| Inter CDFI |  |  | < 0.001 |  |  | 0.005 |
| No | 20 (34) | 21 (17) |  | 6 (27) | 12 (21) |  |
| Little | 26 (45) | 37 (30) |  | 10 (45) | 14 (25) |  |
| Moderate | 9 (16) | 12 (10) |  | 5 (23) | 7 (12) |  |
| Obvious | 3 (5) | 54 (44) |  | 1 (5) | 24 (42) |  |
| Shape |  |  | 0.755 |  |  | 1 |
| Oval or Round | 3 (5) | 9 (7) |  | 1 (5) | 3 (5) |  |
| Irregular | 55 (95) | 115 (93) |  | 21 (95) | 54 (95) |  |
| Margin |  |  | 0.144 |  |  | 0.054 |
| Circumscribed | 12 (21) | 14 (11) |  | 7 (32) | 7 (12) |  |
| Not Circumscribed | 46 (79) | 110 (89) |  | 15 (68) | 50 (88) |  |
| Internal Echo |  |  | 0.117 |  |  | 0.062 |
| Ultra-hypoechoic | 19 (33) | 52 (42) |  | 6 (27) | 30 (53) |  |
| Hypoechoic | 39 (67) | 67 (54) |  | 16 (73) | 25 (44) |  |
| Others | 0 (0) | 5 (4) |  | 0 (0) | 2 (4) |  |
| Posterior Echo |  |  | 0.017 |  |  | 0.032 |
| Shadowing | 17 (29) | 57 (46) |  | 4 (18) | 28 (49) |  |
| No posterior features | 27 (47) | 32 (26) |  | 13 (59) | 19 (33) |  |
| Enhancement | 14 (24) | 35 (28) |  | 5 (23) | 10 (18) |  |

Note. Except where indicated, data are numbers of women with percentages in parentheses. AUS report: BCs status reported by axillary ultrasound; CDFI: color doppler flow imaging.

**Table S4.** The formulas used to calculate these PA radiomics features

***Task One: Differentiation of HER2-Zero Cancers from HER2-Low or HER2-PositiveCancers***

label = 0.7468879668049794 + +0.011219 * intra_exponential_ngtdm_Strength -0.052689 * intra_lbp_3D_m2_glrlm_ShortRunLowGrayLevelEmphasis +0.008357 * intra_logarithm_firstorder_Median +0.049958 * intra_wavelet_HHH_glszm_SizeZoneNonUniformityNormalized -0.035292 * intra_wavelet_HLH_glcm_SumSquares -0.016407 * intra_wavelet_LHH_glszm_ZoneEntropy +0.013399 * intra_wavelet_LLH_glrlm_LongRunHighGrayLevelEmphasis -0.031678 * intra_wavelet_LLH_glrlm_ShortRunHighGrayLevelEmphasis +0.030673 * peri_lbp_3D_k_firstorder_Range +0.016327 * peri_lbp_3D_m1_glszm_GrayLevelVariance -0.024584 * peri_lbp_3D_m1_glszm_SmallAreaHighGrayLevelEmphasis -0.000956 * peri_lbp_3D_m2_glszm_HighGrayLevelZoneEmphasis +0.017851 * peri_wavelet_HHH_firstorder_Skewness -0.007891 * peri_wavelet_HHL_firstorder_Median -0.017420 * peri_wavelet_HLL_firstorder_InterquartileRange +0.038309 * peri_wavelet_HLL_firstorder_Kurtosis +0.010780 * peri_wavelet_HLL_glcm_Idn -0.063008 * peri_wavelet_LHL_glcm_Correlation +0.003680 * peri_wavelet_LLH_firstorder_Maximum

***Task Two: Differentiation of HER2-Low Cancers from HER2-Positive Cancers***

label = 0.2843601895734609 + +0.016800 * intra_lbp_3D_m2_glrlm_ShortRunLowGrayLevelEmphasis +0.001101 * intra_squareroot_glrlm_ShortRunLowGrayLevelEmphasis +0.011824 * intra_wavelet_HLH_gldm_DependenceNonUniformityNormalized -0.007796 * intra_wavelet_HLH_gldm_LargeDependenceHighGrayLevelEmphasis +0.030023 * intra_wavelet_HLH_glszm_ZoneEntropy -0.044515 * intra_wavelet_HLL_glcm_Correlation -0.023433 * intra_wavelet_LHH_glcm_JointEntropy +0.007313 * intra_wavelet_LHH_glszm_GrayLevelNonUniformity +0.073228 * intra_wavelet_LHH_glszm_LowGrayLevelZoneEmphasis +0.005195 * intra_wavelet_LHH_glszm_SmallAreaLowGrayLevelEmphasis +0.001751 * intra_wavelet_LHL_firstorder_Mean -0.014123 * intra_wavelet_LLL_gldm_LargeDependenceLowGrayLevelEmphasis +0.058362 * peri_exponential_glcm_Correlation +0.070741 * peri_gradient_glcm_Idmn +0.033264 * peri_lbp_3D_m2_glrlm_RunVariance +0.019646 * peri_lbp_3D_m2_ngtdm_Busyness -0.009922 * peri_logarithm_ngtdm_Strength -0.057136 * peri_original_shape_Elongation +0.020064 * peri_square_gldm_LargeDependenceHighGrayLevelEmphasis +0.000657 * peri_wavelet_HHH_glszm_LargeAreaLowGrayLevelEmphasis -0.066061 * peri_wavelet_HLH_glszm_ZoneVariance +0.010337 * peri_wavelet_LHH_glcm_Imc1 -0.014734 * peri_wavelet_LLL_ngtdm_Busyness

***Task There: Differentiation of HER2-Zero Cancers from HER2-Low Cancers***

label = 0.6795580110497239 -0.003899 * intra_lbp_3D_m2_gldm_SmallDependenceLowGrayLevelEmphasis -0.094469 * intra_lbp_3D_m2_glrlm_ShortRunLowGrayLevelEmphasis -0.012789 * intra_logarithm_glrlm_ShortRunLowGrayLevelEmphasis +0.045937 * intra_original_shape_Elongation -0.060325 * intra_wavelet_HLH_gldm_GrayLevelVariance -0.017997 * intra_wavelet_HLH_gldm_LowGrayLevelEmphasis +0.060218 * intra_wavelet_LHH_glszm_SmallAreaEmphasis -0.005144 * intra_wavelet_LHL_firstorder_Skewness +0.012488 * peri_gradient_glrlm_ShortRunLowGrayLevelEmphasis -0.007233 * peri_lbp_3D_k_firstorder_Minimum +0.034213 * peri_lbp_3D_k_firstorder_Range -0.035289 * peri_lbp_3D_m1_glszm_SmallAreaHighGrayLevelEmphasis -0.005251 * peri_lbp_3D_m2_glszm_HighGrayLevelZoneEmphasis -0.018264 * peri_lbp_3D_m2_glszm_SmallAreaHighGrayLevelEmphasis -0.010898 * peri_squareroot_glszm_GrayLevelNonUniformity -0.011731 * peri_wavelet_HHL_glszm_SmallAreaEmphasis +0.002030 * peri_wavelet_HLL_firstorder_Kurtosis -0.033290 * peri_wavelet_HLL_firstorder_Skewness -0.009004 * peri_wavelet_HLL_glszm_SmallAreaEmphasis -0.066814 * peri_wavelet_LHL_glcm_Correlation -0.006464 * peri_wavelet_LHL_glszm_LargeAreaHighGrayLevelEmphasis -0.009499 * peri_wavelet_LLH_glrlm_ShortRunLowGrayLevelEmphasis -0.044133 * peri_wavelet_LLH_glszm_ZonePercentage
